# Arbuscular mycorrhizal fungi reduce potassium, cadmium and ammonium losses but increases nitrate loss under high intensity leaching events

**DOI:** 10.1186/s12870-022-03741-3

**Published:** 2022-07-23

**Authors:** Yan Xiao, Lu Chen

**Affiliations:** College of Agro-grassland Science, Nanjing Agricultural University, 210095 Nanjing, P. R. China

**Keywords:** AMF, Leaching, Rainfall, Nutrient loss, Heavy metal

## Abstract

**Background:**

Nutrients and heavy metals can be lost from soils *via* leaching, and arbuscular mycorrhizal fungi (AMF) can influence these events. Soil column experiments were carried out to examine whether leaching intensity and AMF can alter nutrient and Cd uptake in white clover plants and the extent of their losses through leaching.

**Results:**

The presence of AMF significantly increased shoot and total biomass, as well as increased N, P, Cu and Zn uptake independent of water amount applied; while root P and Cu uptakes were promoted by AMF at any water amount treatments. Higher water amounts led to reductions in total N, K and Zn uptake for AMF-colonized plants in comparison to moderate water amount treatments. In the absence of AMF, white clover at low water amount treatment exhibited maximal root Cd uptake. At high water amount treatments, the presence of AMF significantly decreased leachate volumes and the amount of leached NH_4_^+^, K and Cd while AMF significantly increased the amounts of leached NO_3_^−^.

**Conclusions:**

Overall we found that AMF-colonized white clover plants reduced NH_4_^+^, K and Cd loss from soils but increased the risk of NO_3_^−^ loss under high intensity leaching conditions.

**Supplementary Information:**

The online version contains supplementary material available at 10.1186/s12870-022-03741-3.

## Introduction

Nutrient loss from soils through leaching threatens global ecosystems by decreasing soil fertility and productivity and has a negative environmental impact [1, 2]. Plants play a major role in preventing leaching; thus, enhancement in efficiency of plant nutrient utilization is a measure to reduce the environmental impact of leaching [3, 4]. In addition, solubility and mobility of some toxic elements (e.g., Cd) are high, leading to migration of Cd in the polluted soils through rain [5, 6]. Thus, it was suggested that reduction of Cd migration in soil-plant systems is beneficial for environmental safety [7].

Soil biota was also reported to modulate the effects on nutrient loss from leaching [3]. Terrestrial plants have evolved mechanisms that optimize nutrient acquisition by expanding their nutrient interception areas, which is able to minimize nutrient loss after rainfall leaching [4]. Arbuscular mycorrhizal fungi (AMF) colonized plant root hairs expand hyphal networks into surrounding soils [8, 9]. AMF can change soil physical properties through forming macroaggregates that alter the surface traits [10, 11] and increase the capability of surface adsorption of soils [12]. Plants can also be directly benefited by acquiring P, K, Ca, Mg, Cu, Zn, NH_4_^+^ and NO_3_^−^*via* AMF [13–18]. Overall, AMF act to reduce mineral N and P losses that are linked to increases in efficiency of plant nutrient uptake and stabilization of plant-soil systems [19, 20].

AMF have been shown to promote plant growth and accumulate Cd in Cd-polluted soils by the following mechanisms: (1) increasing antioxidant enzyme activity [21]; (2) elevating plant photosynthesis [22]; (3) increasing mineral nutrient uptake of host plants particularly P [23] and (4) secreting glomalin-related soil proteins [24]. Rainfall also induces heavy metal leaching on the surface layer of contaminated soils [5]. Cd can be absorbed by AMF hyphae intertwined with soil surface particles [11]. Heavy rainfall also results in Cd leaching from the soil surface to bottom layers, while AMF have been demonstrated to reduce Cd leaching in maize-planted soils [7]. However, little is known about how rainfall intensity and AMF affect plant growth and the uptake as well as leaching of both nutrients and Cd at different growth stages.

In the current study, we examined the effects of AMF and leaching intensity on element uptakes, as well as dissolved nutrients and Cd ions leaching in soils planted with white clover (*Trifolium repens* L.). We hypothesized that (1) AMF would increase plant growth and nutrient uptake regardless of rainfall amount, and (2) AMF would reduce dissolved ion leaching especially under high intensity rainfall conditions. We designed soil column experiments to monitor the uptake and leaching dynamics in different mowing and leaching events.

## Results

**Fig. 1:**
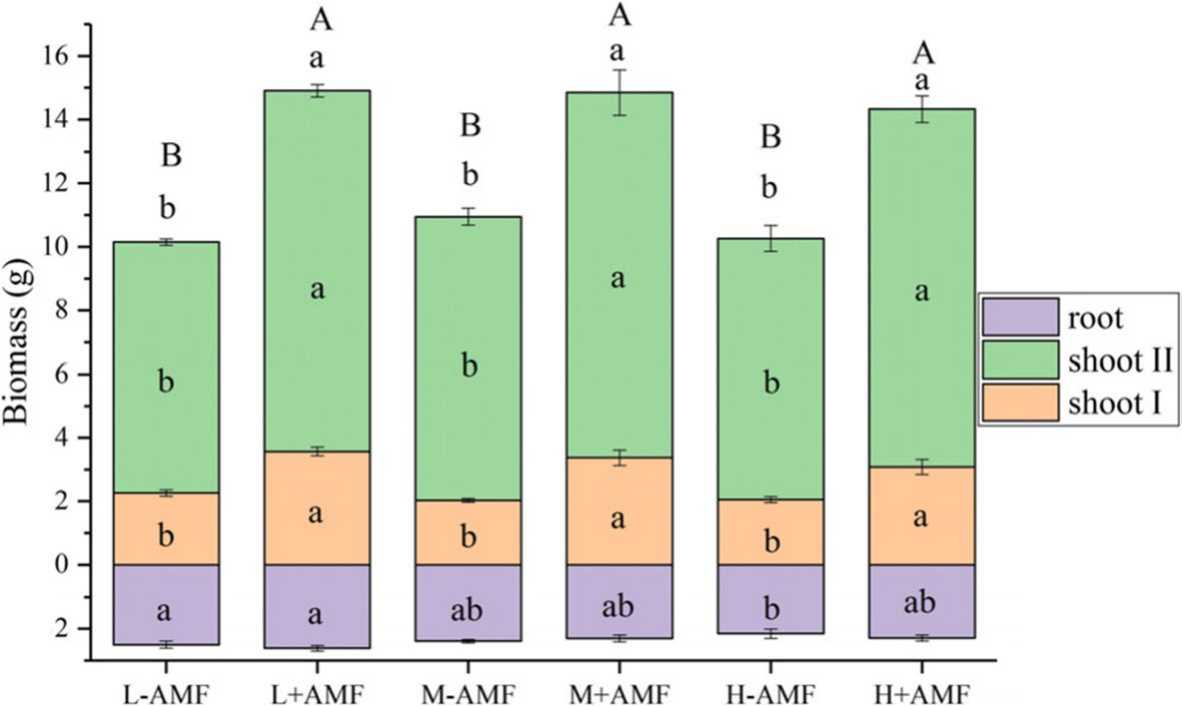
Effects of arbuscular mycorrhizal fungi (AMF) and water amount on plant biomass. Different lower letters denote the significant differences in shoot and root biomass according to Duncan’s post hoc test. Shoot I and II designate plant materials collected at the mowing and harvest stages, respectively. Different upper letters denote the significant differences in total biomass according to Duncan’s post hoc test

**Fig. 2:**
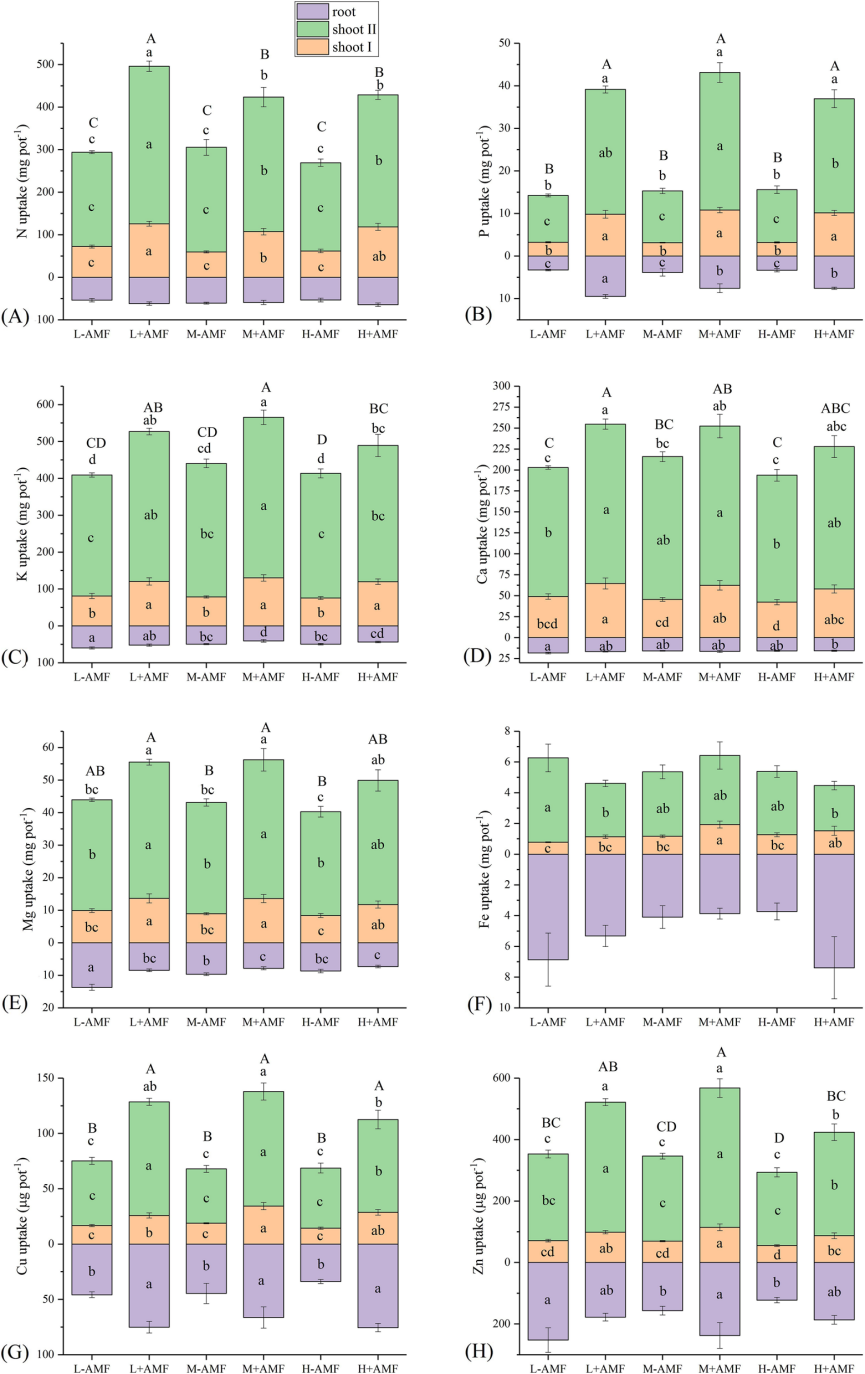
Plant nutrient uptake in different arbuscular mycorrhizal fungi (AMF) and water amount treatments. Different lower letters denote the significant differences in shoot and root nutrient uptake according to Duncan’s post hoc test; Shoot I and II designate plant materials collected at the mowing and harvest stages, respectively. Different upper letters denote the significant differences in total nutrient uptake according to Duncan’s post hoc test. If no significant difference was found among treatments, no letter is indicated

AMF-inoculated plants exhibited significantly higher shoot biomass than plants without inoculation at the mowing and harvest stage, regardless of water amount (Fig. 1). Similarly, N, P, Cu and Zn accumulated in the plant shoots followed a similar trend as plant shoot biomass (Fig. 2A, B, G and H). The root uptake of P and Cu was also improved by AMF (Fig. 2B and G). The shoot uptakes of K, Ca and Mg were increased by AMF only at the mowing stage but these element uptakes in shoots were not elevated by AMF under each leaching event conditions at harvest (Fig. 2C, D and E). The shoot Fe uptake at the mowing stage was only increased by AMF in the moderate water amount treatment (Fig. 2F).

**Fig. 3:**
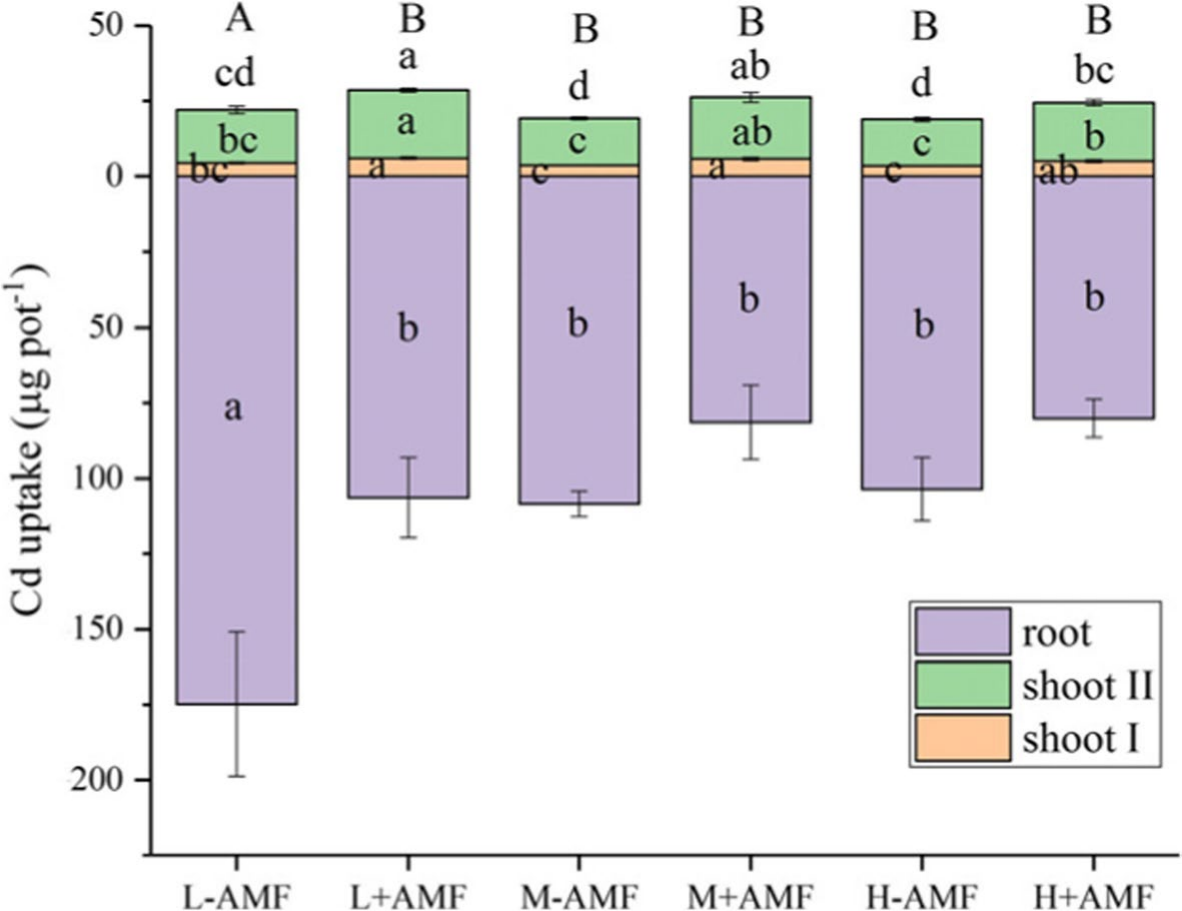
Plant Cd uptakes in different arbuscular mycorrhizal fungi (AMF) and water amount treatments. Different lower letters denote the significant differences in shoot and root Cd uptakes according to Duncan’s post hoc test. Shoot I and II designate plant materials collected at the mowing and harvest stages, respectively. Different upper letters denote the significant differences in total Cd uptakes according to Duncan’s post hoc test

Water treatment amount did not affect shoot biomass at either at mowing or harvest stage (Fig. 1). Moderate water amount significantly reduced root K, Mg, Zn and Cd uptakes in non-AMF plants, whereas only root K uptake was decreased by moderate water amount in AMF plants (Figs. 2 and 3). The total K and Zn uptakes for the AMF-colonized plants for the high water amount treatments were significantly lower than those of moderate water amount treatments (Fig. 2 C and H). L-AMF treatment exhibited maximum Cd uptake in the roots. Nevertheless, shoot Cd uptakes were increased by AMF at the mowing and harvest stages (Fig. 3).

**Fig. 4:**
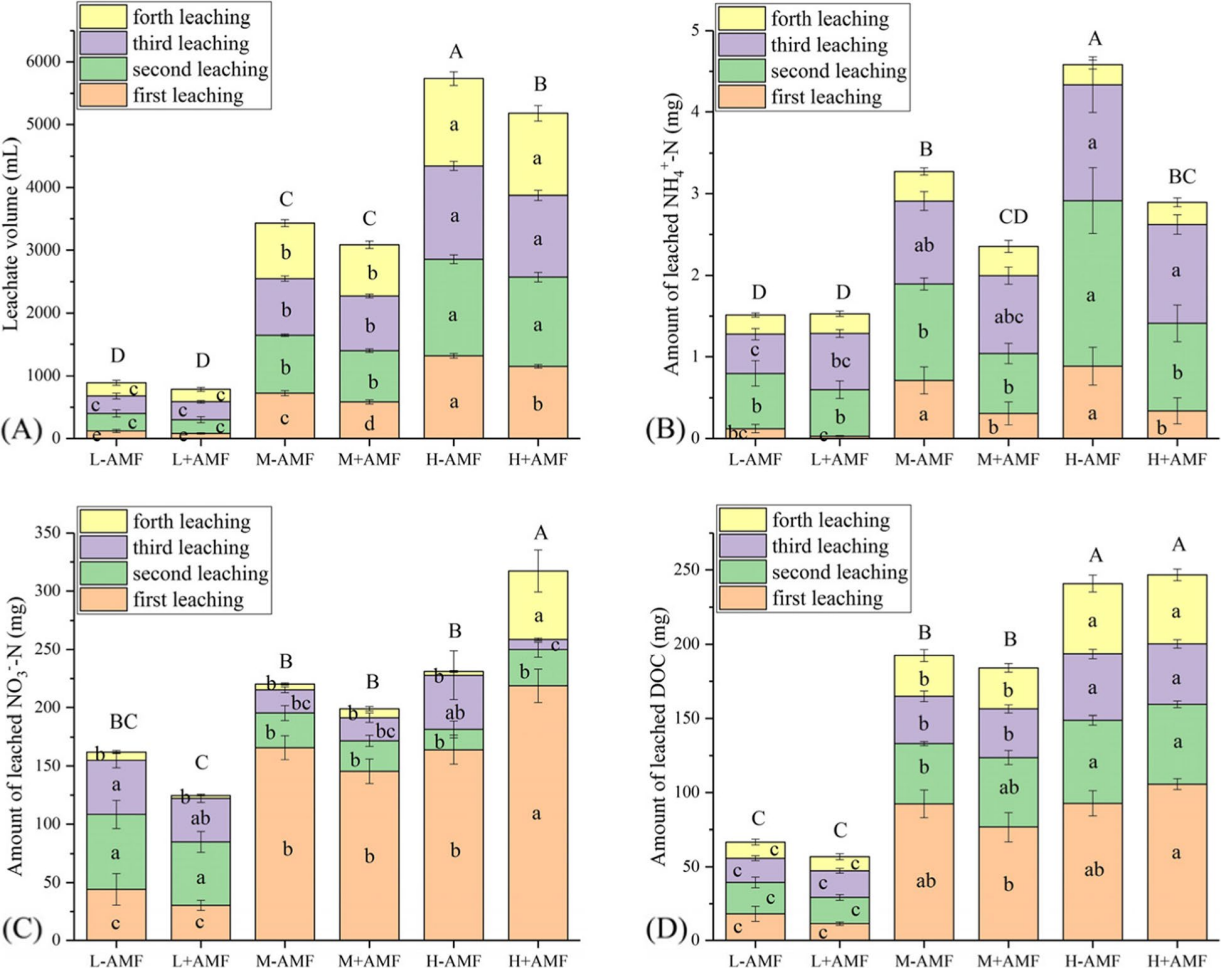
Leachate volume and amount of leached dissolved C and N in different arbuscular mycorrhizal fungi (AMF) and water amount treatments. Different lower letters denote the significant differences in each leaching event according to Duncan’s post hoc test. Different upper letters denote the significant differences in total leachate volume and amount of leached dissolved C and N according to Duncan’s post hoc test. If no significant difference was found among treatments, no letter is indicated

In the moderate and high water amount treatments, AMF resulted in a significant decrease of leachate volumes in the first leaching event. Although leachate volumes were not significantly different in the subsequent second, third and fourth leaching events for the AMF-colonized *versus* non-colonized plants, the sum of leachate volume of the four leaching events was significantly reduced in the presence of AMF when high water amounts were applied (Fig. 4A).

The amounts of NH_4_^+^ found in leachates for the three water amount treatments displayed different patterns. AMF had no influence on the amounts of leached NH_4_^+^ in the low water treatments for every leaching event. In contrast, AMF decreased the amounts of leached NH_4_^+^ only in the first leaching event when moderate water amount was applied. In both first and second leaching events, the amounts of leached NH_4_^+^ were reduced when high water amount was applied. Thus, for the moderate and high water treatments, the total amounts of leached NH_4_^+^ over the 4 leaching events of AMF inoculation treatments were less than these of non-AMF inoculation treatments (Fig. 4B).

The total amounts of leached NO_3_^−^ differed from the trends for NH_4_^+^. In the low and moderate water treatments, the amounts of leached NO_3_^−^ were not affected by AMF for all leaching events. Nevertheless, with high water amount inputs, the presence of AMF significantly increased the amount of leached NO_3_^−^ in the first and fourth leaching events, as well as the total amount of leached NO_3_^−^ over the 4 leaching events. In contrast, AMF significantly decreased the amount of leached NO_3_^−^ in the third leaching event. The DOC in the leachates was only increased by water amount but not by AMF for all leaching events (Fig. 4D).

**Fig. 5:**
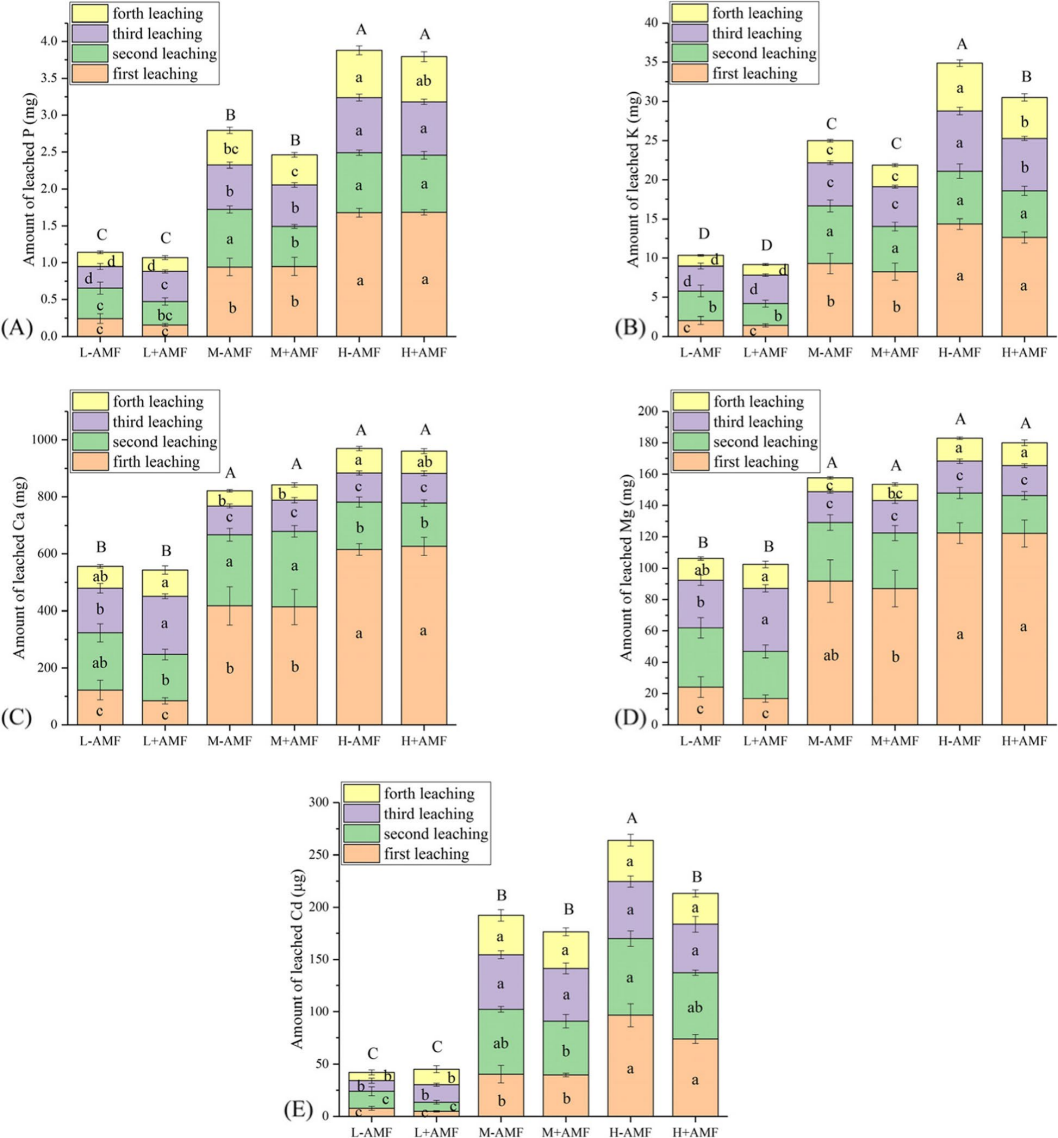
Amount of leached element in different arbuscular mycorrhizal fungi (AMF) and water amount treatments. Different lower letters denote the significant differences in each leaching event according to Duncan’s post hoc test. Different upper letters denote the significant differences in total amount of leached element according to Duncan’s post hoc test. If no significant difference was found among treatments, no letter is indicated

In this study, Fe, Cu and Zn concentrations in leachate were not detected by ICP-OES (data not shown). In the low water treatments, it was found that AMF increased the total amounts of leached P, Ca and Mg only in the third leaching event (Fig. 5A, C, D). In the moderate water treatments, the amounts of leached P, K, Ca and Mg were not significantly altered by AMF except for a reduction of P in the second leaching event (Fig. 5A-D). Compared with the treatments that were not colonized with AMF, the presence of AMF significantly decreased the amount of leached K in the third and fourth leaching events. Reduction of total amount of leached K and Cd in the high water treatment appeared in the AMF inoculation group (Fig. 5B).

**Fig. 6:**
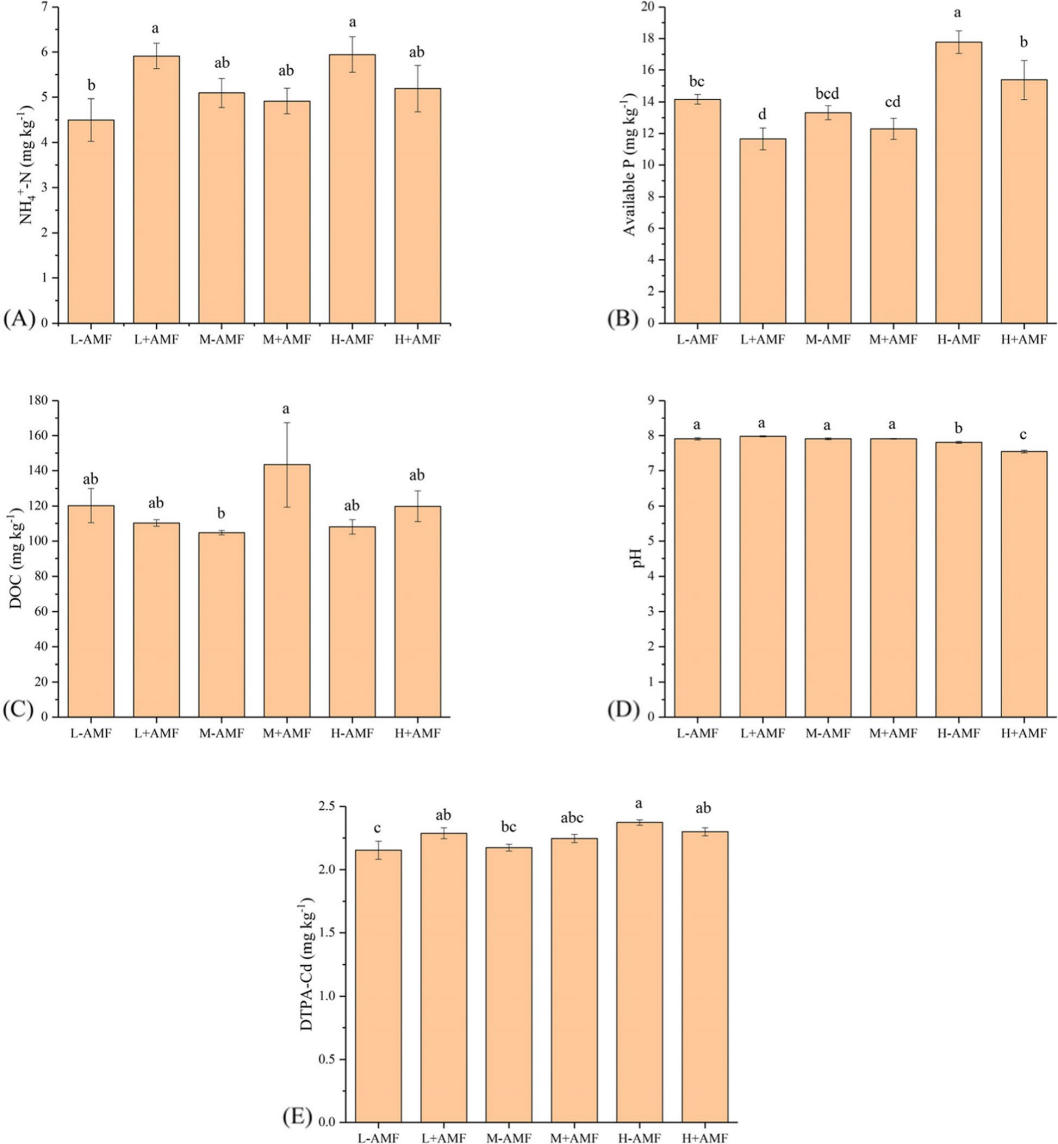
Soil chemical properties in different arbuscular mycorrhizal fungi (AMF) and water amount treatments. Different lower letters denote the significant differences according to Duncan’s post hoc test

It was found that soil DOC concentration in the M + AMF treatment was significantly higher than M-AMF treatment (Fig. 6C). The maximal soil DTPA-Cd concentration was found in the H-AMF treatment group (Fig. 6E). Soil pH was not significantly different for the groups with low and moderate water amount inputs. Compared with L-AMF treatment, pH values for H-AMF and H + AMF treatments decreased 0.1 and 0.36 units, respectively (Fig. 6D).

## Discussion

AMF has been reported to increase host plant biomass [22, 25, 26]. However, not every nutrient or mineral was equally promoted by the presence of AMF [16, 18, 27, 28]. Our results indicated that AMF increased shoot biomass, as well as N, P, Cu and Zn uptake at every growth stage, whereas shoot K, Ca and Mg uptakes were not increased by AMF at every growth stage when leaching intensity increased (Figs. 1 and 2). Therefore, we concluded that the nutrient uptake in response to AMF varied, dependent on the plant growth stage and the type of nutrients. In addition, it was speculated that the reduction of nutrient element uptake did not limit plant shoot growth regardless of water amounts applied. Increased shoot Cd uptake induced by increased biomass inAMF-inoculated plants has been previously reported [16, 18, 27, 28], which might explain the enhancement of Cd uptakes in AMF-inoculated plant shoot from our findings (Fig. 3).

A recent study found that soils containing plant roots had higher non-capillary porosity and saturated hydraulic conductivity than soils without plant roots, which facilitated preferential migration of soil solutions [29]. In this study, we did not observe increases in root biomass following AMF inoculation (Fig. 1), but significant reductions in leachate volumes occurred in the first leaching event when moderate and high water were applied (Fig. 4A). The reasons for this phenomenon may include (1) AMF-colonized plants could facilitate root growth at the early growth stages, (2) a higher water consumption in AMF-colonized plant shoots was accompanied by ahigher biomass, and (3) other unknown root morphological characteristics that were not yet identified in this experiment could also influence leaching.

These results were partly consistent with the hypothesis that AMF can reduce soil NH_4_^+^ loss, especially under conditions of increased rainfall intensity (Fig. 4B). Similar NH_4_^+^ losses that occurred *via* leaching have been reported in other studies [13, 20, 30]. AMF colonized plants can absorb N as amino acids [31], NO_3_^−^ [26] or NH_4_^+^ [32]. Reduction in N losses *via* leaching has also been linked to the promotion of plant growth, N assimilation and NH_4_^+^ immobilization in soils involving AMF-root systems in pasture soils [13]. In addition, reduction of leachate volume also contributes to decreases of leached N [20, 33, 34]. In our study, increased plant N uptake and reduction of leachate volumes both contributed to the reductions of NH_4_^+^ loss through leaching (Figs. 2 A, 4A).

Previous studies have demonstrated that NH_4_^+^ is preferentially absorbed over NO_3_^−^ in mycorrhizal plants [32, 35]. In particular, AMF exert their effects on N leaching for NH_4_^+^ but not for NO_3_^−^ [13]. In contrast, neither AMF nor water volume influenced the amount of leached NH_4_^+^ whereas NO_3_^−^ loss through leaching was less for AM-colonized plants as compared with non-colonized plants when water regime was regular [36]. In red clover mycorrhizal grassland microcosms, the amount of leached NO_3_^−^ may also be associated with the N fixation by *Rhizobia spp*., resulting in N loss *via* leaching [4, 37]. The amounts of leached NO_3_^−^ in our experiments were greater for AMF-colonized plants with high water treatments (Fig. 3 C). It was predicted that an enhancement of N fixation in the presence of AMF may influence the available N pool in soil and lead to variable levels of NO_3_^−^ loss by leaching in our study. AMF can alter soil microbial communities including those involved in N-cycling processes, which could lead to N losses *via* denitrification as previously reported [4, 38, 39]. N leaching from plant-soil systems induced by rainfall events is influenced by numerous factors including soil characteristics, plant root systems, soil organisms and their interactions [1]. Therefore, the levels of N fixation, soil N-cycling microorganisms and nitrification processes that existed in our experimental plant-root systems may have influenced NO_3_^−^ loss in different ways at different growth stages.

Plant roots and AMF hyphae both produce large amounts of exudates that are released into soils, including organic carbon derived from photosynthesis [10]. Root exudates such as the low-molecular weight malic, succinic and citric acids were enhanced by AMF-colonization of soils contaminated by heavy metals [40]. However, our results indicated that AMF did not influence the dynamics of DOC losses (Fig. 4D).

Generally, AMF promote P uptake in host plants [14] and reduce leached P in soils in normal rainfall events [13, 41]. However, we found that P uptake in plants was enhanced by AMF at each of water treatment groups (Fig. 2B), but the total P lost by leaching was unaltered by the presence of AMF regardless of water amount (Fig. 5A). Nevertheless, at low water amount treatments, the presence of AMF significantly increased the total amount of leached P, Ca and Mg in the third leaching event (Fig. 5A, C, D). The total amount of leached P, K, Ca and Mg increased with water amount increased (Fig. 5). All of these results indicated that nutrient leaching is dependent on plant growth stages, rainfall intensity, nutrient type, soil nutrient levels and instantaneous plant nutrient uptake status.

Plant root systems can directly take up nutrients and heavy metals, thereby lowering these ion concentrations in soils [42]. Maize roots could decrease concentration of Cd in the interflow while had no effect on leached Cd from the contaminated soils; whereas AMF could reduce concentration of Cd in the interflow and in the leachate [7]. The role of AMF played in Cd uptake could be explained by changes in soil macroaggregates and total glomalin-related soil protein (T-GRSP) content in Cd-contaminated soils [7, 43]. Additionally, soil macroaggregates possessing relatively higher mass Cd loading reduced Cd mobility and its export from contaminated soils [44]. However, the presence of AMF did not result in increase in total Cd uptake in white clover plants in our experiments (Fig. 3). Therefore, Cd uptake in plants may not be the primary reason for the reduction in the amount of leached Cd when high water treatment was applied. Although AMF reduced soil pH that might increase the risk of heavy metal mobilization, the soil available Cd was not affected by AMF in the high water treatment group (Fig. 6). We only measured the soil available Cd before plant harvest, thus, more information regarding the dynamics of soil available Cd is needed in the future to explain the reduction of Cd loss *via* leaching.

## Conclusions

The increased shoot biomass, as well as uptake of N, P, Cu and Zn in AMF-colonized white clover plants partly support the hypothesis that AMF may increase plant growth and nutrient uptake regardless of rainfall amount. Reduced losses of soil NH_4_^+^, K and Cd induced by AMF occurred under high intensity rainfall conditions. Opposite to our assumption, AMF significantly reduced NH_4_^+^, K and Cd loss from soils but increased soil NO_3_^−^ loss when high intensity leaching occurred. The changes of soil in different growth stages will help us to further clarify the mechanism of rainfall on plant element absorption and soil ion leaching in future studies.

## Materials and methods

### Substrate and microbial inoculum propagation

The location of soil collection was Baima Town of Nanjing, Jiangsu Province, China. The soils were mixed with sand (2:1) and then sterilized by at 120 °C in an autoclave. The chemical properties of mixtures used for this study were listed in Table S1. A mixture of AMF inocula containing *Glomus aggregatum*, *G. etunicatum*, *G. intraradices*, *G. tortuosum* and *G. versiforme* was used in this experiment. Cadmium chloride solution was added into soil and mixed well. Cd concentration of the mixtures was 3 mg Cd kg^− 1^. The mixture was maintained in the greenhouse for six months before use.

### Experimental design

The experiments in this study included three water amount treatments (i.e., low (L), moderate (M) and high (H)) and two AMF treatments (i.e., plants lacking AMF (–AMF) and plants inoculated with (+ AMF)), resulting in 6 different treatments (i.e., L–AMF, L + AMF, M–AMF, M + AMF, H–AMF and H + AMF) and each treatment was conducted in replicates. The variety of white clover used in this experiment is haifa. White clover seeds were sown in pots (35 cm in height and 16 cm in diameter) filled with 6 kg of substrate soil on November 26, 2018. In the + AMF treatments, white clover was inoculated with 200 g of mycorrhizal fungi whereas 200 g sterilized inoculants used for AMF propagation were added in the –AMF treatments. To maintain microbial communities, each of all treatments received 50 mL soil microbial filtrate after screening through a nylon mesh (25 μm). Twenty-five days after sowing, the clover was thinned to 15 seedlings per pot. On April 7, 2019, the plants were cut to 10 cm, which was designated as the mowing event. Plants were harvested by hand approximately on May 13, 2019, and this was designated the harvest event. During the growth period, leaching events were carried out on March, 9, March 18, April 9 and April 19, 2019, respectively. In each leaching event, deionized water were applied in the low, moderate and high water amount treatments (i.e., 720, 1440 and 2160 mL), respectively. Drainage holes at the bottom of the pots were used to collect the leachate.

### Measurements

Clover shoot biomass and elemental uptake were calculated as the sum of biomass or element uptake in the mowing and harvest events. The elemental uptake was calculated as concentrations in the plant tissues multiplied by the biomass. Total biomass was the sum of shoot and root biomass, and total element uptake was the sum of shoot and root element uptake. Total leachate volume and total amount of element loss was the sum of leachate volume or the amount of element loss from all four leaching events. Mycorrhizal colonization was determined as previously described [45].

Analytical measurements of P, K, Ca, Mg, Fe, Cu, Zn and Cd were conducted using inductively coupled plasma-optical emission spectrometry (ICP-OES). Clover samples were digested in a nitric:perchloric acid mixture (3:1 v/v) [27]. Total N in plant tissues was determined after digestion in H_2_SO_4_:H_2_O_2_ solution and its concentrations were determined by spectrophotometry as previously described [46]. Diethylenetriamine pentaacetic acid (DTPA) was used to extract Cd from soils as previously described [47]. NH_4_^+^ was extracted using 0.5 M K_2_SO_4_ and measured using the indophenol blue method. Dissolved organic carbon (DOC) levels in soils were quantified with a TOC-TN analyzer as previously described [48]. NH_4_^+^, DOC and elements in leachates were also determined as described above. NO_3_^−^ was measured using a dual wavelength method as previously described [48]. Soil pH was determined using a pH meter in the supernatant of a soil:deionized water mixture (in 1:5 ratio). Available P in soils was measured by the molybdenum blue method as previously described [49].

### Statistical analyses

Statistical analyses were conducted using SPSS version 13.0 (IBM, Chicago, Ill, USA). Duncan multiple comparisons were used to compare differences among different treatments.

## Supplementary Information

**Additional file 1.**

## Data Availability

The data will be available on request by contacting corresponding author.

## Abbreviations

AMF: arbuscular mycorrhizal fungi
N: nitrogen
P: phosphorus
K: potassium
Ca: calcium
Mg: magnesium
Fe: Ferrum
Cu: copper
Zn: Zinc
Cd: cadmium
DOC: dissolved organic carbon
DTPA: diethylenetriamine pentaacetic acid
