# Supplementary material for: Arbuscular mycorrhizal fungi reduce potassium, cadmium and ammonium losses but increases nitrate loss under high intensity leaching events
Source: BMC Plant Biol. 2022 Jul 23;22:365. doi: 10.1186/s12870-022-03741-3 (PMC9308228; doi:10.1186/s12870-022-03741-3)
Supplement: Supplementary file 1 — Additional file 1. [file 12870_2022_3741_MOESM1_ESM.docx]

**Supplementary materials**

Table S1 Chemical properties of mixtures used for this study.

| Properties |  |
| --- | --- |
| pH | 7.51 |
| Soil organic carbon (g kg^-1^) | 3.58 |
| Total nitrogen (g kg^-1^) | 0.95 |
| Total phosphorus (g kg^-1^) | 0.24 |
| Total potassium (g kg^-1^) | 5.81 |
| Total magnesium (g kg^-1^) | 3.82 |
| Total calcium (g kg^-1^) | 3.74 |
| Available K (mg kg^-1^) | 262.19 |
| Available P (mg kg^-1^) | 18.54 |
| NH_4_^+^-N (mg kg^-1^) | 2.45 |
| NO_3_^‾^-N (mg kg^-1^) | 37.59 |

**
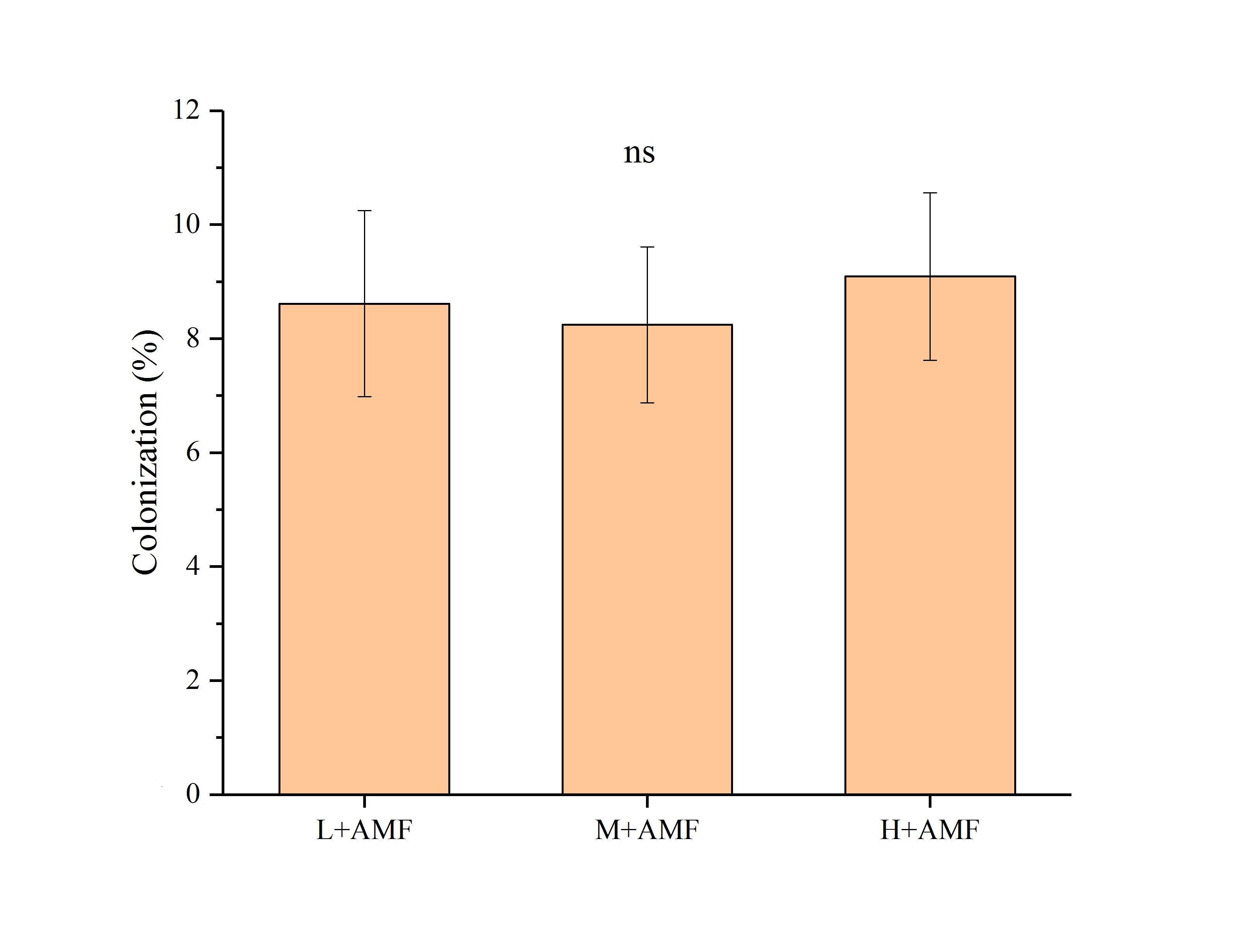
**

Fig. S1 Effects of water amount on root mycorrhizal colonization.


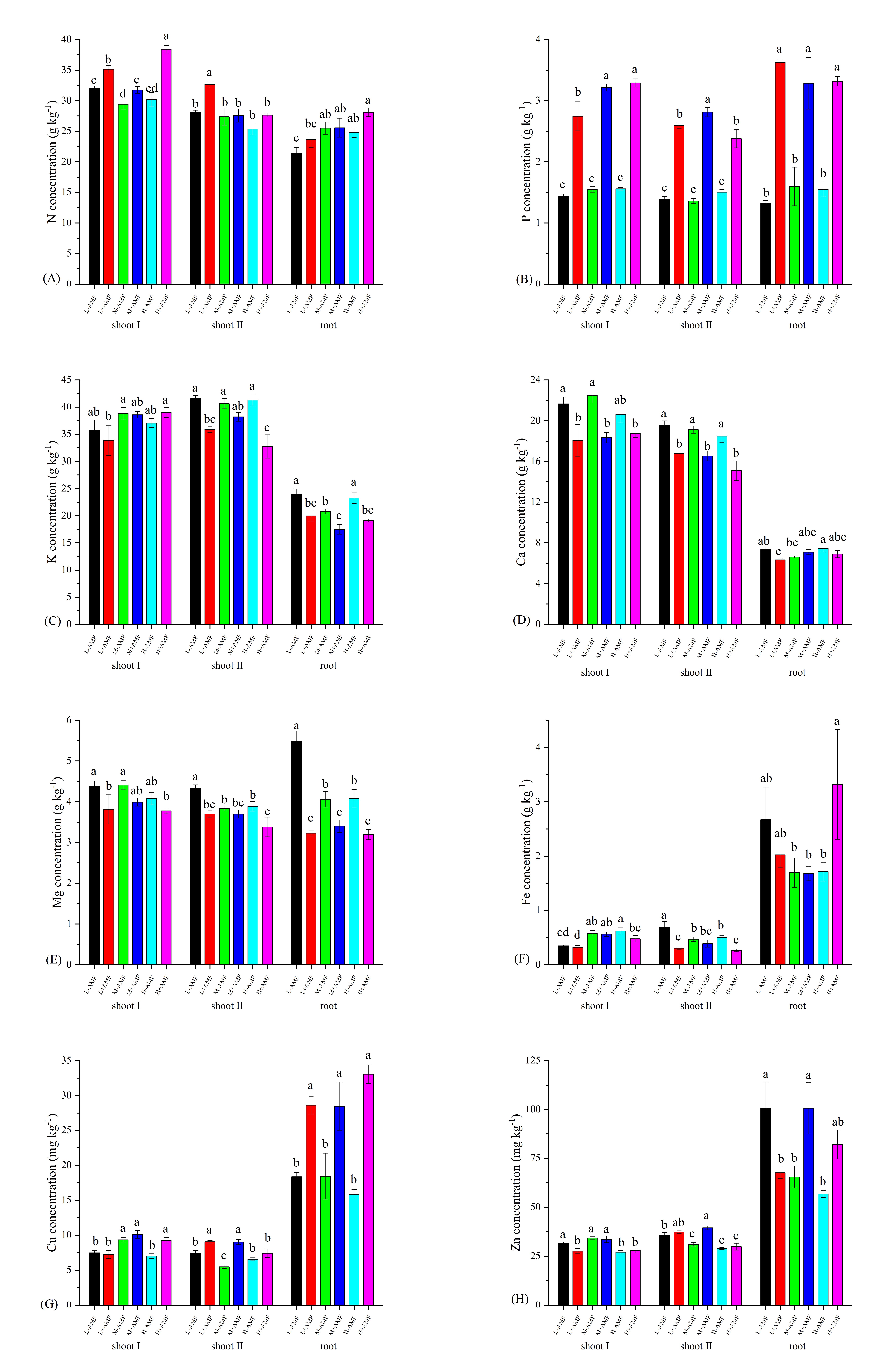


Fig. S2 Plant nutrient concentration in different arbuscular mycorrhizal fungi (AMF) and water amount treatments. Different lower letters denote the significant differences in shoot and root nutrient uptake according to Duncan’s post hoc test; Shoot I and II designate plant material collected at the mowing and harvest stages, respectively.


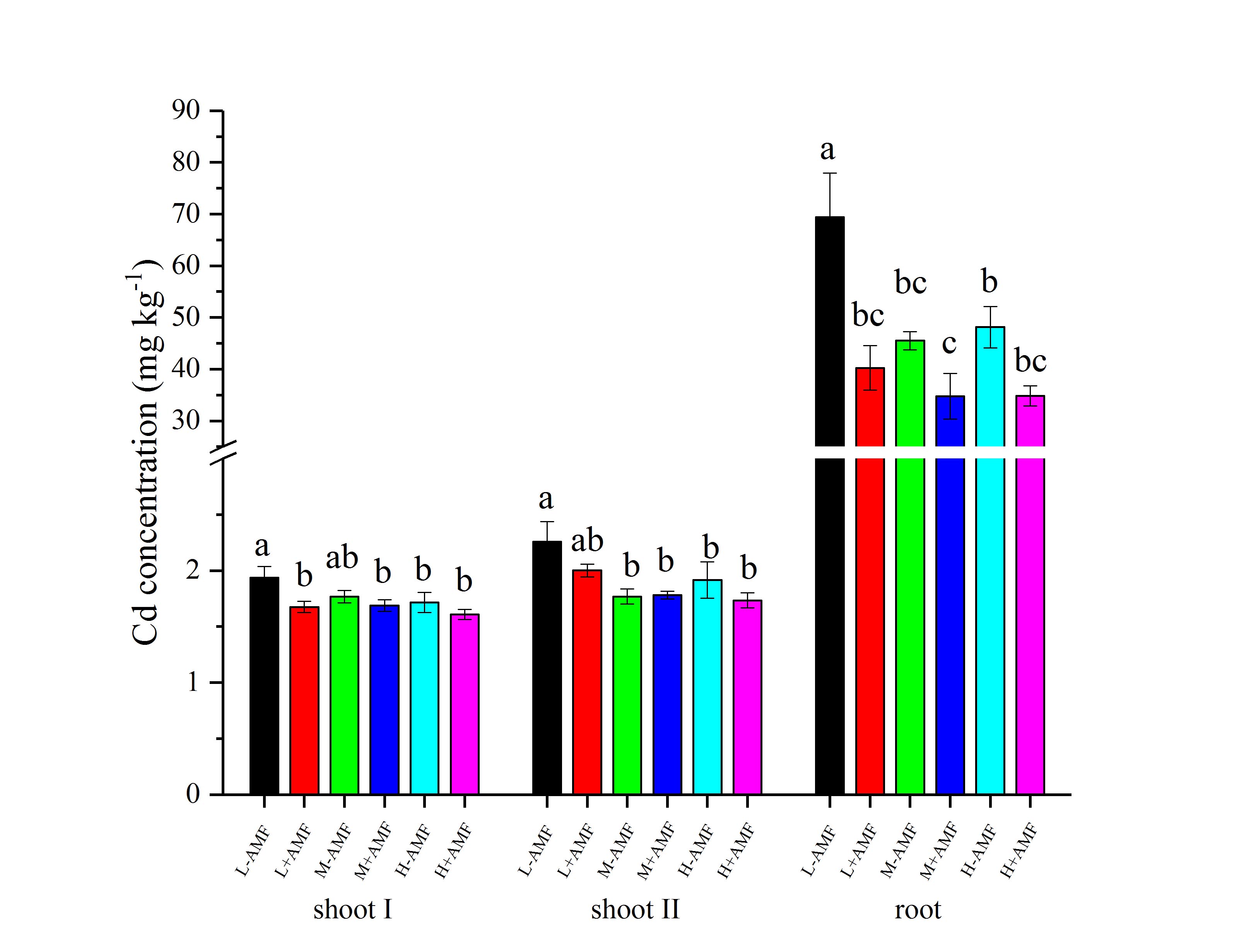


Fig. S3 Plant Cd concentrations in different arbuscular mycorrhizal fungi (AMF) and water amount treatments. Different lower letters denote the significant differences in shoot and root Cd concentration according to Duncan’s post hoc test. Shoot I and II designate plant material collected at the mowing and harvest stages, respectively.


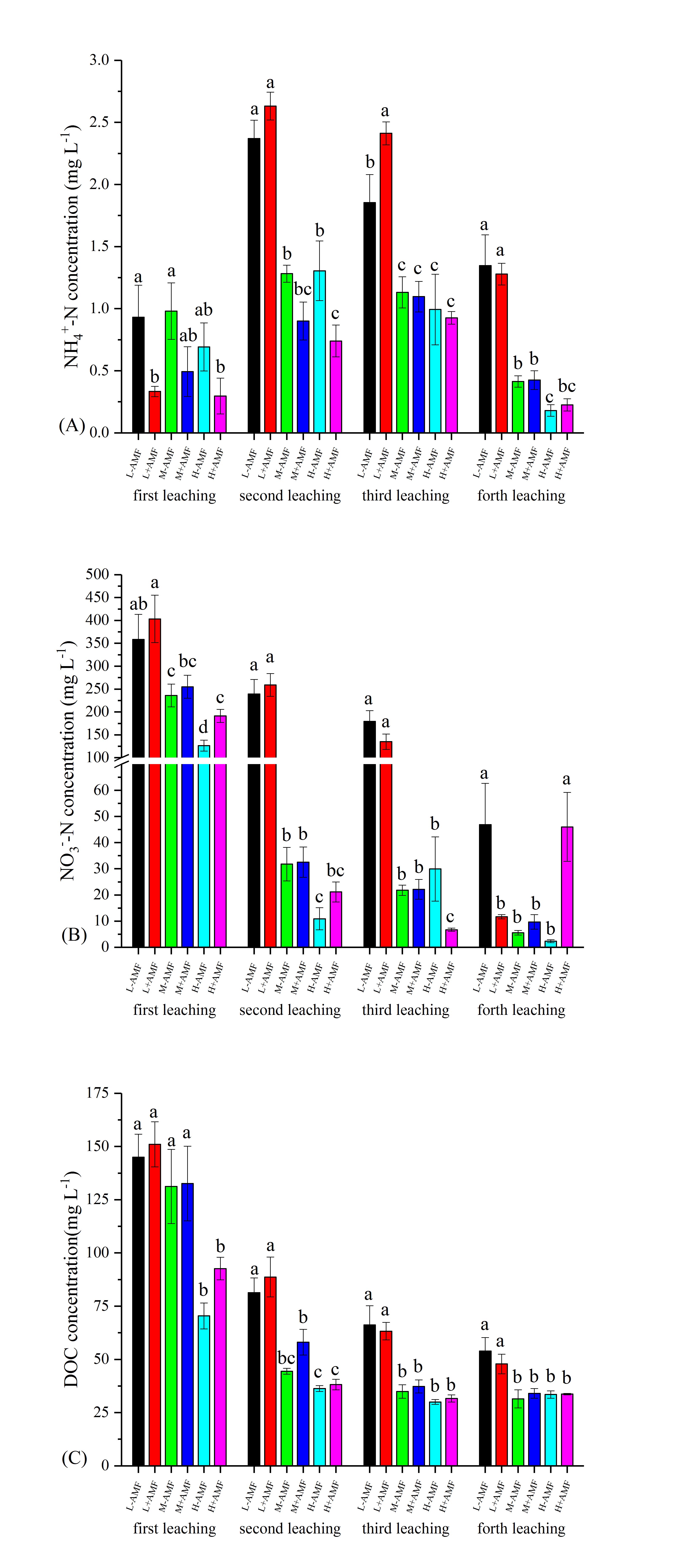


Fig. S4 Concentrations of leached dissolved C and N in different arbuscular mycorrhizal fungi (AMF) and water amount treatments. Different lower letters denote the significant differences in each leaching event according to Duncan’s post hoc test.


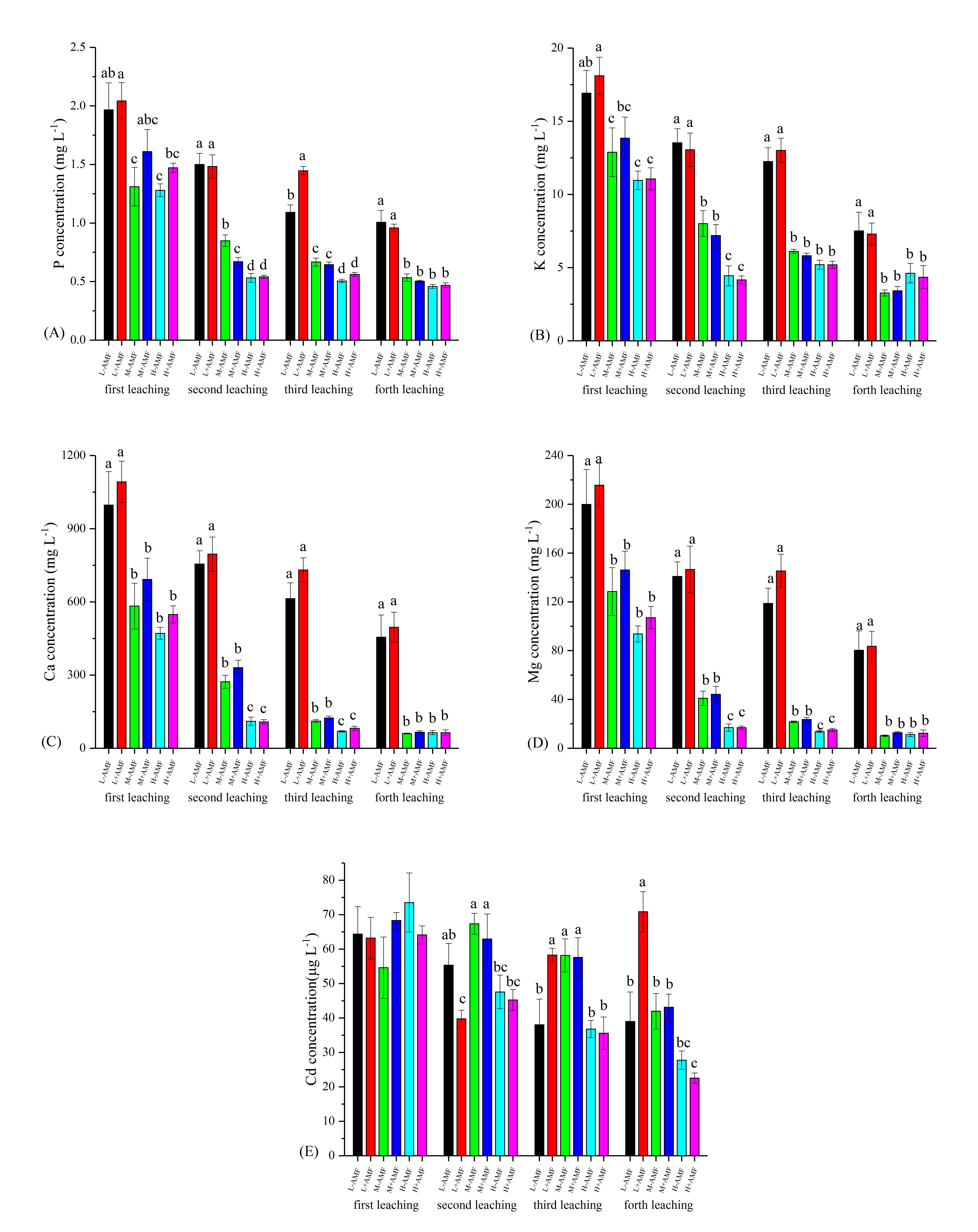


Fig. S5 Concentrations of leached element in different arbuscular mycorrhizal fungi (AMF) and water amount treatments. Different lower letters denote the significant differences in each leaching event according to Duncan’s post hoc test.
